# 

**Published:** 1911-01

**Authors:** 


PUBLISHED MONTHLY. ATLANTA SUBSCRIPTION $1.00

### JOURNAL-RECORD OF MEDICINE

Successor to { Atlanta Medical and Surgical Journal, Established 1855. and Southern Medical Record, Established 1870. } 56th Year

Vol. LVI. ATLANTA, GA., JANUARY, 1911. No. 10

